# Risks to Human Health from Mercury in Gold Mining in the Coastal Region of Ecuador

**DOI:** 10.3390/toxics12050323

**Published:** 2024-04-29

**Authors:** Carlos Mestanza-Ramón, Samantha Jiménez-Oyola, Juan Cedeño-Laje, Karla Villamar Marazita, Alex Vinicio Gavilanes Montoya, Danny Daniel Castillo Vizuete, Demmy Mora-Silva, Luis Santiago Carrera Almendáriz, Santiago Logroño-Naranjo, Guido Mazón-Fierro, Renato Herrera-Chávez, Giovanni D’Orio, Salvatore Straface

**Affiliations:** 1 Research Group YASUNI-SDC, Escuela Superior Politécnica de Chimborazo, Sede Orellana, El Coca EC-220001, Ecuador; demmy.mora@espoch.edu.ec (D.M.-S.); israel.logronio@espoch.edu.ec (S.L.-N.); 2 Department of Environmental Engineering, University of Calabria, 87036 Rende, Italy; salvatore.straface@unical.it; 3 Facultad de Ingeniería en Ciencias de la Tierra, ESPOL Polytechnic University, Escuela Superior Politécnica del Litoral (ESPOL), Campus Gustavo Galindo, km 30.5 Vía Perimetral, P.O. Box 09-01-5863, Guayaquil EC-090101, Ecuador; sjimenez@espol.edu.ec (S.J.-O.); juanced@espol.edu.ec (J.C.-L.); karmavil@espol.edu.ec (K.V.M.); 4 Faculty of Natural Resources, Escuela Superior Politécnica de Chimborazo, Panamericana Sur, Km 1 ½, Riobamba EC-060155, Ecuador; vinicio.gavilanes@espoch.edu.ec (A.V.G.M.); danny.castillo@espoch.edu.ec (D.D.C.V.); 5 Faculty of Science, Escuela Superior Politécnica de Chimborazo, Panamericana Sur, Km 1 ½, Riobamba EC-060155, Ecuador; luissantiago.carrera@espoch.edu.ec; 6 Faculty of Business Administration, Escuela Superior Politécnica de Chimborazo, Panamericana Sur, Km 1 ½, Riobamba EC-060155, Ecuador; guido.mazon@espoch.edu.ec; 7 Facultad de Ciencias Políticas y Administrativas, Universidad Nacional del Chimborazo, Av. Antonio José de Sucre Km 1 ½ Vía a Guano, Riobamba EC-060155, Ecuador; renato.herrera@unach.edu.ec; 8 Department of Economics, Statistics and Finance, University of Calabria, 87036 Arcavacata di Rende, Italy; giovanni.dorio@unical.it

**Keywords:** risk assessment, non-carcinogenic risk, gold mining, water quality, environmental pollution

## Abstract

Artisanal and small-scale gold mining (ASGM) plays a crucial role in global gold production. However, the adoption of poor mining practices or the use of mercury (Hg) in gold recovery processes has generated serious environmental contamination events. The focus of this study is assessing the concentration of Hg in surface waters within the coastal region of Ecuador. The results are used to conduct a human health risk assessment applying deterministic and probabilistic methods, specifically targeting groups vulnerable to exposure in affected mining environments. Between April and June 2022, 54 water samples were collected from rivers and streams adjacent to mining areas to determine Hg levels. In the health risk assessment, exposure routes through water ingestion and dermal contact were considered for both adults and children, following the model structures outlined by the U.S. Environmental Protection Agency. The results indicate elevated Hg concentrations in two of the five provinces studied, El Oro and Esmeraldas, where at least 88% and 75% of the samples, respectively, exceeded the maximum permissible limit (MPL) set by Ecuadorian regulations for the preservation of aquatic life. Furthermore, in El Oro province, 28% of the samples exceeded the MPL established for drinking water quality. The high concentrations of Hg could be related to illegal mining activity that uses Hg for gold recovery. Regarding the human health risk assessment, risk values above the safe exposure limit were estimated. Children were identified as the most vulnerable receptor. Therefore, there is an urgent need to establish effective regulations that guarantee the protection of river users in potentially contaminated areas. Finally, it is important to continue investigating the contamination caused by human practices in the coastal region.

## 1. Introduction

Mercury (Hg) is one of many metals whose natural distribution has been profoundly altered by human activities. It can be found in the environment in various chemical forms that lack biological and physiological functions in living organisms, having detrimental effects. Consequently, exposure to this element is of toxicological concern [1,2]. Studies indicate that artisanal and small-scale gold mining (ASGM) emits about 880 tons of mercury annually [3,4,5]. This endeavor has been consistently undertaken as a means to alleviate poverty and promote socio-economic development [6,7], producing between 10% and 15% of the world’s mined gold [8,9]. In many countries, ASGM is characterized by the use of rudimentary low-tech methods with inefficient gold recovery [10]. Generally, the activity is associated with negative impacts from the use of mercury (Hg), which causes extensive environmental degradation during and after the development of mining activities [11]. 

The process involves burning the amalgam to vaporize mercury (Hg) and retrieve the gold [12]. Without adequate precautions, capture devices, or protective gear during this procedure, mercury vapors are released into the air [13]. Numerous studies indicate that mercury from both natural and human-induced sources is progressively becoming a significant cause for global concern regarding human health, the aquatic environment, and the food chain. This underscores the importance of addressing environmental mercury pollution [14,15,16,17]. 

This element ranks third among the most hazardous elements to human health, according to the U.S. Government’s Agency for Toxic Substances and Disease Registry (ATSDR) [18]. It is estimated that approximately 19 million people worldwide face health risks due to mercury exposure [19]. In 2015, more than 450 sites around the world were identified by the Toxic Sites Identification Program as locations where mercury exposure poses a threat to the health of the population [20]. Gradual accumulation in both aquatic and terrestrial food chains leads to significant exposure to humans. Due to its ability to travel over long distances, mercury can be detected even in remote areas [21,22].

For decades, artisanal and small-scale gold mining (ASGM) has been a well-established core economic activity in Ecuador [23]. In Ecuador, severe environmental impacts caused by mining activities have been reported between 1998 and 2023, such as the contamination of air, surface waters, soils, and sediments [24,25,26,27,28,29,30,31,32]. In this regard, our research aims to complement the cited studies and provide updated information on the degree of mercury contamination in mining environments.

There are gold processing centers, particularly in the south of the country, that operate in a responsible manner, integrating cleaner extraction and processing techniques. In contrast, there are still mineral processing plants that use Hg illegally [33]. This unregulated and extensive use of mercury is a typical feature of illegal mining, causing the pollution of surface waters and an escalation in sediment load [34]. The lack of proper regulation led to a troubling increase in illegal mining across the country, particularly evident in the San Lorenzo canton in the province of Esmeraldas [35].

The effects of mercury on human health have been investigated by a number of scholars [36]. These have shown that Hg contamination has become one of the most serious problems threatening human subsistence [37,38]. Mercury (Hg) is recognized as one of the most harmful metals with potential impacts on human health due to its volatility, its long life in the atmosphere, and its tendency to accumulate in living organisms [39]. Human poisoning can result from both short-term and prolonged exposure to mercury [40]. Areas affected by mining activities introduce ecological threats to aquatic ecosystems, water systems, and result in harmful health effects for humans, animals, and plants [41,42]. 

Documented effects include adverse impacts on the reproductive and immune systems, as well as an increased risk of cardiovascular disease and premature death in cases of significant exposure [43]. The main routes of surface water exposure for humans are incidental (or not) water ingestion and dermal contact [44]. Understanding how to limit mercury (Hg) pollution and identifying appropriate strategies for preventing adverse health consequences associated with Hg emissions is crucial [45]. 

The quantification of potential risks linked to human exposure to specific contaminants is the primary objective of the widespread utilization of health risk assessment [46]. In the case of mercury, it is crucial to understand the various harmful impacts on human health depending on the route of exposure [15,47]. Ingestion of mercury can cause kidney damage, neurotoxicity, gastrointestinal disorders, and affect the development of the central nervous system, especially in fetuses exposed during pregnancy [48]. Inhalation of mercury vapors can cause lung irritation, acute or chronic neurotoxicity, cardiovascular effects, and immunosuppression [49]. In addition, dermal exposure to mercury can cause dermatitis, skin sensitization, and systemic absorption, with effects similar to mercury ingestion [50]. Emphasizing the importance of properly addressing mercury exposure is critical to prevent adverse health effects and protect human health in general [51].

Both deterministic and probabilistic methods can be utilized for estimating health risk assessment [52]. The deterministic approach presents the resultant health risk as a single-point value [53]. In contrast, probabilistic risk assessment produces the risk output as a range of values, integrating the probability distribution of various input parameters in the risk equation [54,55]. This approach furnishes information that supports decision-makers by providing a quantitative estimate of risk and aids in the allocation of resources to control exposure to environmental hazards. Risk assessment requires public communication of the possible sources of contaminants, as well as the levels of contamination and risk to the environment and people’s health; This information is crucial to promote the implementation of preventive measures and appropriate management policies for the protection of public health [56].

On the other hand, it is important to mention that the research presents an innovative approach in examining the risks to human health from mercury in gold mining in the coastal region of Ecuador. Unlike previous research that may have addressed this issue more generally, our research focuses specifically on this unique geographic region, allowing us to better identify and understand the specific challenges and implications in this context.

The main objective of this study is to evaluate the concentration of mercury (Hg) in fresh surface water within the gold mining regions located in the coastal zone of Ecuador. In addition, it seeks to analyze the potential risk to human health associated with these concentrations by applying deterministic and probabilistic methods. The information provided in this study constitutes a baseline for future research and serves as an input for the formulation of sound management strategies aimed at the reduction in environmental contamination in vulnerable areas. 

## 2. Materials and Methods

### 2.1. Study Area

**Figure 1:**
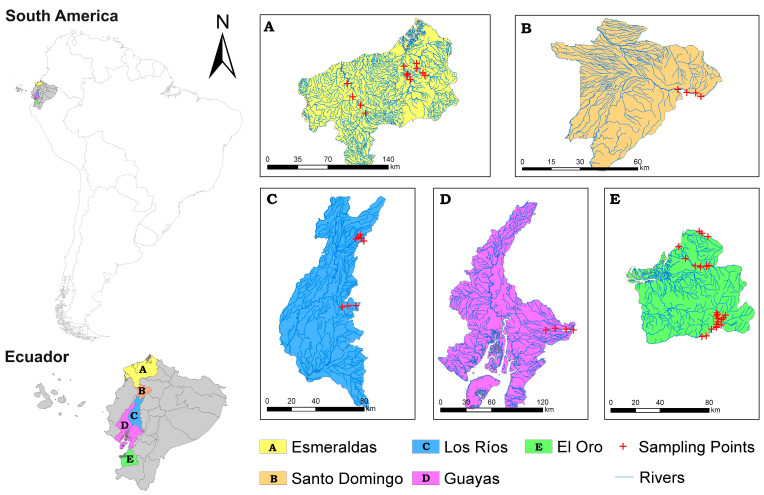
Study area and location of the sampling sites.

The study focused on five provinces of the Ecuadorian coastal region also known as the littoral region: Esmeraldas, Santo Domingo, Los Ríos, Guayas, and El Oro (Figure 1), with an approximate population of 8.5 million people [57]. The primary economic activities in the area include agriculture, forestry, and mining. The latter, particularly through the use of mercury for gold recovery, has adverse environmental implications [58,59]. In the coastal region, there is a mining activity regulation in force that includes the definition/delimitation of primary extraction zones, alluvial extraction zones, and mineral processing zones. The provinces of the coastal region with gold mining areas in each exploitation stage in descending order are: El Oro (n = 231), Esmeraldas (n = 12), Guayas (n = 7), Santo Domingo (n = 3), and Los Ríos (n = 2) [60].

### 2.2. Sampling and Laboratory Analysis

The field investigation was conducted in the first semester of 2022 during the rainy season, between March and June, during which rainfall usually ranges between 200 and 350 mm [38]. A total of 54 water samples were collected from water bodies (rivers and streams) connected to or crossing mining concessions (Figure 1). Samples were taken from the following rivers: Santiago, Cayapas, and Esmeraldas (Esmeraldas); Pilatón (Santo Domingo); Mangulita, Mangulita Grande, and Oncebí (Los Rios); Chimbo (Guayas); Jubones, Fermín, Amarillo, Calera, and Pindo (El Oro). In the water sampling process, 250 mL amber bottles were used; these samples were acidified by adding 0.10 mL of nitric acid. In order to apply an optimal sampling process, quality policies, code of ethics, and confidentiality regarding the transportation of the samples were strictly considered. All samples were guarded from the sampling point to the Science Laboratory of the Escuela Superior Politécnica de Chimborazo, Sede Orellana, Ecuador. The next step was to determine the concentration of Hg. This process was carried out using an atomic absorption and hydride generation method (Atomic Absorption Spectrophotometry). Throughout the analysis, the Standard Methods, Ed. 23. 2017, 3112B—Acid digestion: EPA method 3015, 2007 was used as a reference. Prior to the analysis, the total samples were prepared by applying the nitric acid digestion procedure described by the U.S. Environmental Protection Agency (EPA) method 7473 [61,62]. The whole process was subjected to quality control considering the NIST 1640a certified reference material and the Hg recovery percentages ranged from 88% to 97%.

### 2.3. Health Risk Assessment 

It was considered that adults and children alike can be exposed to Hg through ingestion or dermal contact with polluted water, as they do not have access to drinking water. Therefore, the non-carcinogenic (systemic) risk for adults and children was calculated for the residential and recreational scenarios. Both scenarios were considered because, in rural areas, rivers/streams are essential supplies of water, albeit untreated, for domestic consumption and are widely used, mainly by children and youth, for recreational purposes. The Hg doses received (ADD) through ingestion and dermal contact were calculated based on the methodology proposed by the United States Environmental Protection Agency (USEPA, 2001, 2004), according to Equations (1) and (2).

(1) $${ADD}_{ingestion}=\frac{C\times EF\times IR\times ED}{AT\times BW}$$

(2) $${ADD}_{dermalcontact}=\frac{C\times EF\times ET\times ED\times SA\times kp}{AT\times BW}$$

where C (mg/L) is the concentration of Hg in water; EF (days/year) is the exposure frequency; IR (L/day) is the water ingestion rate; ED (years) is the duration of exposure; ET (hours/event) is the exposure time; SA (cm^2^) is the surface area of exposed skin; kp (0.001 cm/hour) is the skin permeability constant; AT (days) is average exposure time (365 days × ED); and BW (kg) represents the body weight of the receptors. The non-carcinogenic risk (HQ) for adults and children was calculated using Equation (3) for both residential and recreational scenarios.

(3) $${HQ}_{\frac{oral}{derm}}=\frac{{ADD}_{\frac{oral}{derm}}}{{RfD}_{\frac{oral}{derm}}}$$

where ADD is the dose received via the oral or dermal exposure route; RfD is the reference dose (RfDoral = 0.0003 mg/kg-day and RfDdermal = 0.000021 mg/kg-day) (USDoE, 2022). The hazard index (HI) for each exposure scenario was calculated by summing the HQingestion plus the HQdermal contact. HQ > 1 and HI > 1 indicate potential adverse health effects to receptors.

To minimize the high uncertainty of the risk assessment, probabilistic risk analysis was used [54,55], along with traditional deterministic analysis [53]. The parameters (given as point values and/or probability distributions) employed in this study are presented in Table 1. Geographic Information Systems (GIS) environment (ArcGis 10.8.2 software) was used for the analysis and processing of the spatial information and for the generation of the point risk maps in the deterministic method. In addition, the R programming language (R Core Team 2019) was used for statistical analysis of the data and probabilistic risk simulation. A total of 1000 iterations were performed to ensure the stability of the probabilistic analysis.

### 2.4. Statistical Analysis and Data Processing

Statistical analysis was performed using Statgraphics software, version 19. Descriptive statistics were utilized to examine trends present in the data set. The normality of the data distribution was assessed with the Kolmogorov–Smirnov test. For the processing of spatial information and the generation of maps, the Geographic Information System, software ArcMap 10.8.1, was used.

## 3. Results

### 3.1. Hg Concentration in Water

**Figure 2:**
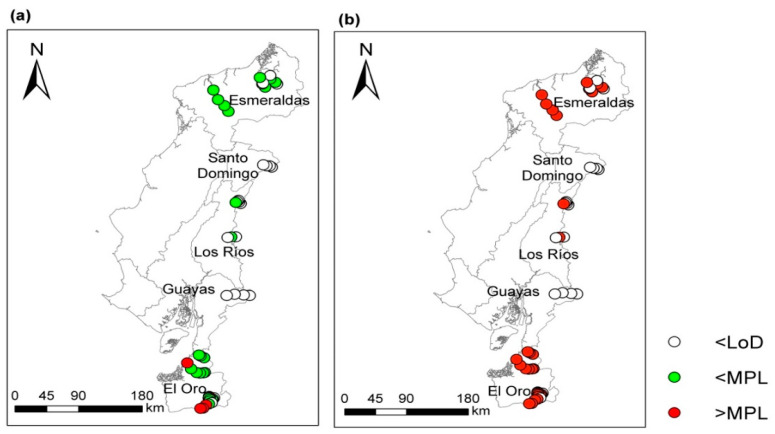
Sampling points and Hg concentration. Results compared with the maximum permissible limit (MPL) according to the Ecuadorian Water Quality Guidelines for (**a**) drinking water and (**b**) for the preservation of aquatic life.

Table 2 presents the Hg concentration in the water samples by province. It is highlighted that 37% of the samples analyzed were below the detection limit (<0.0005 mg/L). The data regarding Hg concentrations were compared considering the Ecuadorian standard (INEN 1108) on the protection of human health in drinking water (0.006 mg/L). Likewise, the admissible quality criteria for the preservation of aquatic life and wildlife in fresh, marine, and estuarine waters (0.0002 mg/L) (TULSMA, 2015) were considered (Figure 2). Additionally, Hg values were compared to the regulation of Canadian drinking water quality (0.001 mg/L).

In the Northern Ecuadorian coastal regions, Hg concentration decreased in the following order: El Oro > Esmeraldas > Los Ríos > Santo Domingo > Guayas. The results obtained are related to the intensity of mining activities in each province. In El Oro, gold mining activity takes place mainly in the cantons of Zaruma, Portovelo, Atahualpa, and Piñas, which constitute the Zaruma–Portovelo mining district, where the largest gold and silver processing capacity in southern Ecuador is located, with a production capacity of between 20 and 300 tons/year. In El Oro, mineral extraction is primordial, that is, through mining galleries. This area has been widely questioned due to the use of Hg in the gold recovery processes [33], where it is estimated that approximately 0.66 tons/year of Hg are released into the environment. In the province of Esmeraldas, mining activity predominates in the cantons of Eloy Alfaro and San Lorenzo, on the Santiago and Bogota rivers, respectively; here, mining is of the alluvial type, on riverbanks and riverbeds. It is estimated that the amount of Hg emitted into the atmosphere is approximately 2.54 tons/year [60]. On the other hand, as expected, the provinces of Los Ríos, Santo Domingo, and Guayas, which have little gold mining activity, showed lower levels of Hg contamination in the rivers analyzed.

The results show that the concentration of Hg in the province of El Oro was in the range of 0.0007–0.0965 mg/L; 12% of the samples analyzed in this province were below the LoD, while 20% exceeded the MPL for drinking water quality. Regarding the freshwater quality, 88% of the samples exceed the MPL. These results agree with previous studies that reported as the province of El Oro has been severely affected by illegal mining practices, inadequate management of mining waste, and indiscriminate use of Hg in mineral beneficiation activities, mainly in the Zaruma–Portovelo Mining District [26,27,68].

In Esmeraldas, the concentration of Hg ranged from 0.0005 mg/L to 0.045 mg/L. In total, 100% of the samples were below the MPL for drinking water quality but 75% of the samples exceeded the MPL for freshwater quality; the other 25% were below the LoD. Esmeraldas, like the province of El Oro, has been severely affected by illegal mining that occurs mainly in the riverbeds and has caused a high concentration of potentially toxic elements, constituting a risk to the ecosystem [44]. 

In the province of Los Ríos, the Hg content varied between 0.0005 and 0.0023 mg/L. With respect to drinking water, all samples were within the established limit, but 33% failed to meet the quality criteria for the preservation of aquatic life and 67% of the samples were below the LoD. To our knowledge, there are no studies that report on the degree of impact on surface water resources due to mining practices in the Los Ríos area. So, this paper constitutes a baseline for future research in the area, given that the high concentrations of Hg represent a potential risk to the environment. In Santo Domingo and Guayas, the Hg concentration at all sampled points was below the detection limit of the analytical method. This may be because in Santo Domingo metallic mining activity, mainly gold mining, has not had a great development as in Guayas, where the predominant mining activity is non-metallic mining and the exploitation of aggregates and stone. 

Our results are in the range of Hg concentrations in rivers, reported in previous research carried out in Ecuador. In these studies, the Hg values were in the range of 0.0005–0.009 mg/L [69] and 0.005–0.011 mg/L [70] in the Ecuadorian Amazon, and in the range of 0.0006–0.09 mg/L in the Andean region of Ecuador [71]. Furthermore, in certain areas, contamination may have contaminated groundwater bodies, as reported in the Hg contents measured in wells in the Ecuadorian Amazon, with Hg concentrations between 0.0007 and 0.0056 mg/L [72]. The values where the highest Hg contents were reported correspond to regions with important mining activity.

The Hg concentrations reported in this study correspond to the rainy season; however, it is expected that in the dry season the contents of the contaminants will increase because the dilution process decreases. Therefore, it is crucial to carry out continuous monitoring of water quality in this area.

### 3.2. Human Health Risk Assessment

#### 3.2.1. Deterministic Approach

**Figure 3:**
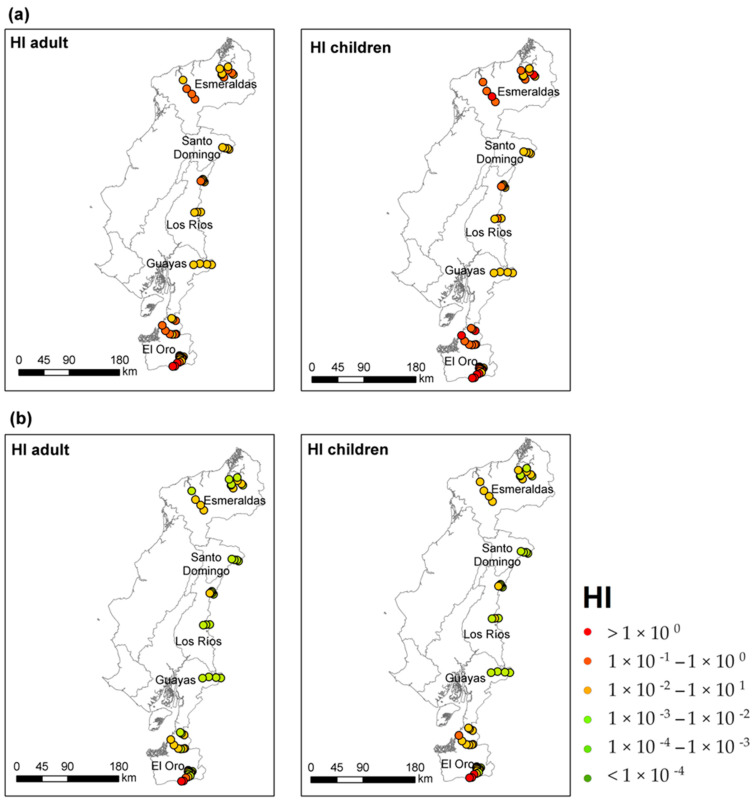
Hazard Index (HI) for receptors exposed to contaminated surface water in the Ecuadorian coastal zone for the following scenarios: (**a**) residential and (**b**) recreational.

The results of the deterministic risk assessment are presented in Figure 3 as point risk maps. In addition, Table 3 summarizes the deterministic HQ and HI values for the 95th percentile of Hg exposure for each province assessed and for each receptor. When Hg concentrations were below the limit of detection (LoD) of the measurement equipment, the LoD/2 value was used for risk calculation. 

The HI values by province followed the decreasing order: El Oro > Esmeraldas > Los Ríos > Santo Domingo > Guayas. HI values > 1 were reported only in the provinces of El Oro and Esmeraldas. Regarding the recreational scenario, three of the sampled sites presented concentrations above the acceptable risk threshold (HI > 1) for adult and child receptors (Figure 3); these concentrations from the sampled sites were located in the province of El Oro. Surface water sampling was chosen because not everyone in the country has access to drinking water, a situation that especially affects indigenous communities and rural areas. Regarding the residential scenario, 15 of the sampled sites had concentrations above the safe exposure threshold for receptors, mainly for children. Sites with HI > 1 were determined in Esmeraldas and El Oro, provinces where the highest Hg concentration was detected. Water has been identified as the main exposure pathway in the residential scenario for El Oro and Esmeraldas provinces. 

Ingestion HQ values ranged between 2.26 × 10^−2^ and 8.74 × 10^0^ for adult receptors and between 6.56 × 10^−2^ and 2.53 × 10^+1^ for children, with children being the most exposed receptors, as the risk values for children are three times the risk values determined for adults, this is a consequence of the lower body mass. On the other hand, the dermal contact route presented very low HQ values in the residential scenario, varying between 8.02 × 10^−4^ and 3.10 × 10^−1^ for adult receptors and between 1.17 × 10^−3^ and 4.52 × 10^−1^ for children; thus, it is considered that this route of exposure is not significant and does not contribute to the risk. Our findings are consistent with previous studies that have reported that in residential settings, water ingestion is the main route of entry of potentially toxic elements into the human body in contaminated areas [24,28,44,69,73]. 

In contrast, in the recreational scenario, intake did not pose a risk to receptors in any of the provinces evaluated, with values ranging from 1.38 × 10^−5^ to 7.07 × 10^−3^ for adults and 1.44 × 10^−4^ to 5.54 × 10^−2^ for children. However, in the recreational scenario, dermal contact exceeded the safe exposure threshold for both receptors in El Oro province; HQ_dermal_ values in the study area were in the range of 2.73 × 10^−3^ and 1.05 for adults and 3.98 × 10^−3^ and 1.54 for children. These results were to be expected since the water intake ratio during recreational activities (swimming or playing in rivers) is very low, with intake values (IR) of 0.053 and 0.090 m/L for adults and children, usually (USEPA, 2011). On the other hand, the skin is highly exposed to contaminants during recreational activities, even more so in warm places, as is the coastal regions of Ecuador, where the use of rivers is quite common, mainly in rural communities. The trend of the recreational scenario risk results are in agreement with the findings reported by [29] in the Ecuadorian Amazon, where Hg through dermal contact in the recreational scenario represented an elevated risk to receptors.

#### 3.2.2. Probabilistic Approach 

**Figure 4:**
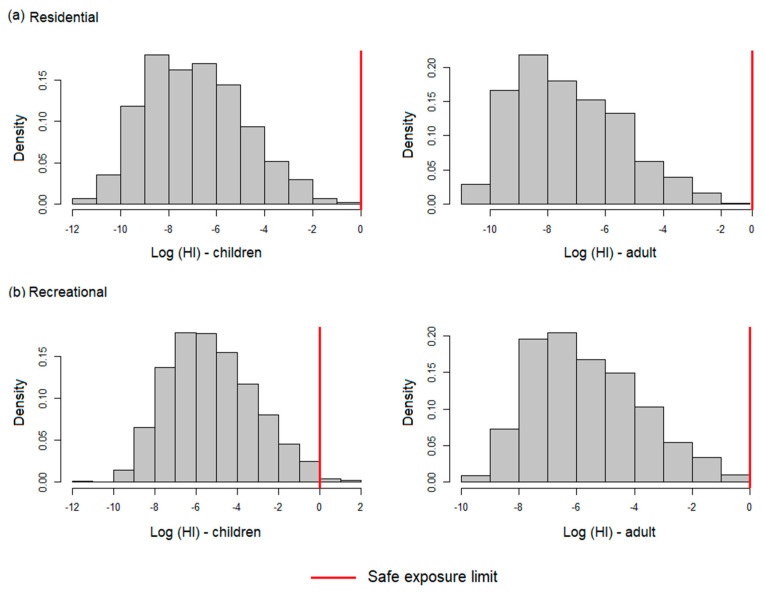
Histogram of HI related to Hg in contaminated surface waters for recipient adults and children in (**a**) residential and (**b**) recreational settings. HI values are given in logarithm.

The results of the risk estimated by the probabilistic method yielded acceptable risk values for adult and child receptors in the residential scenario (Figure 4a), with a maximum value of HI_adults_ = 1.74 × 10^−1^ and HI_children_ = 8.71 × 10^−1^. Children are the most vulnerable receptors, with HI values up to five times higher than in adults, because of their lower body mass. In the recreational scenario (Figure 4b), the maximum estimated HI value for child receptors was 4.13, exceeding four times the safe exposure threshold, while for adults the risk value remained below the acceptable limit (maximum HI for adults = 8.69 × 10^−1^ In the residential setting, water ingestion was the main route of exposure. In this sense, there is a possibility that users of rivers in the northern coastal regions, where high concentrations of Hg have been detected, may develop adverse health effects. The safe exposure threshold for children in the recreational setting was exceeded at the 99th percentile, meaning that 1% of child receptors are exposed to developing adverse health effects as a consequence of Hg exposure. Therefore, a risk communication strategy should be implemented to limit the exposure of river users and keep risk levels within acceptable limits.

The results of the deterministic analysis differ from the results of the probabilistic analysis; however, the information obtained with both methods can complement each other and improve the interpretation of the results obtained, as well as provide greater inputs for the analysis of decision makers. The main difference detected is that, according to the probabilistic methodology, the residential scenario does not generate risk for the receptors, while, according to the deterministic methodology, both the residential and recreational scenarios generate risk at localized sites in the study area. In this context, based on a conservative criterion, the use of rivers located in contaminated mining environments is not recommended, since long-term exposure to contaminants such as Hg could affect the health of its users. Furthermore, it is important to consider that in areas of illegal gold mining, there are, in addition to Hg, other potentially toxic elements that could generate adverse effects on the ecosystem and the population: a detailed study of other pollutants such as arsenic, cadmium, chromium, lead, etc., is recommended, since these elements have been detected in other gold mining areas of Ecuador [30,31,32,73]. Deterministic and probabilistic analysis approaches are considered appropriate to adequately account for data uncertainty. A study in PLACE reported by Sherman et al. (2015) notes that, in most ASGM settings, women and children are actively involved in ASGM activities and therefore often face health- or life-threatening exposures.

## 4. Discussion

The study reveals a concerning scenario regarding the presence of mercury in the northern coastal regions of Ecuador, as mercury contamination can pose harmful effects on both the environment and people’s health. Elevated mercury concentrations were detected in the provinces of El Oro and Esmeraldas, situated in the southwest and northwest of the analyzed region, surpassing the limits established by Ecuadorian regulations. The situation concerning the preservation of aquatic life is particularly alarming concerning water quality. In this regard, the wildlife in the coastal region would encounter a significant impact, potentially threatening both ecosystems and the most vulnerable species in the area.

Mercury concentration in 63% of the samples exceeded the maximum permissible limit (MPL) set by Ecuador’s water quality standards for freshwater; the other 37% were below the LoD. Additionally, samples from El Oro (20%) were found to exceed the maximum permissible limit (MPL) for drinking water. Furthermore, according to Canadian water quality guidelines [74], 44% of the samples exceeded the allowable limit of 0.001 mg/L. It is imperative to address mercury contamination and implement measures to safeguard the health of the exposed population [22,75]. Elevated mercury concentrations of up to 4.60 µg/L in surface water samples (rivers, streams, mining ponds, springs) highlight the potential transfer of mercury into the aquatic food chain. Mercury tends to accumulate in the fatty portions of fish tissues and, through the food chain, can enter the liver, posing a significant risk to human health [76]. 

Numerous studies have reported that environmental impacts caused by mining activity manifest through air, water, soils, crops, and sediments, subsequently affecting the health of people exposed to pollutants. It is essential to formulate a comprehensive strategy and appropriate regulations to mitigate the adverse effects of mining on the environment and promote sustainable practices in the industry. Increased environmental regulation has been shown to limit the ecological impacts of mining, although these impacts may vary regionally. Additionally, mining activities often involve extensive land excavation, deforestation, and displacement of natural ecosystems, resulting in habitat degradation and decreased biological diversity. Hence, implementing actions to mitigate these effects and encourage responsible and sustainable mining practices is crucial.

One of the primary factors contributing to the increase in illegal mining, closely linked to environmental contamination, is the inadequate supervision and regulation of mining activities by the state. 

In Ecuador, there is legislation that criminalizes unauthorized exploitation of mineral resources, financing or supply of machinery for illegal extraction, and negative environmental impact, known as the Organic Comprehensive Penal Code. This legislation imposes both criminal and administrative measures on those who fail to comply with mining and environmental regulations. This legal instrument is crucial primarily for its ability to promote the regulation and supervision of mining activity in the country and encourage sustainable and responsible practices in the mining industry [77]. Despite the existence of this legal tool, there has not been a significant decrease in illegal mining in the country due to weak law enforcement, influenced by technicians’ fear of violence associated with illegal gold mining practices.

It is fundamental to consider norms and laws guiding activities toward environmentally, economically, and socially sustainable development. Therefore, establishing regulations for managing environmental impacts left by mining activities to protect the environment and mining community inhabitants is necessary.

Over time, the inability of state regulations to effectively regulate and monitor mining activities, coupled with the presence of armed groups, limited sustainable economic alternatives for local communities, minimal community participation in decision-making, and high international demand for minerals, have contributed to the persistence of illegal mining. Addressing these issues comprehensively is crucial to foster equitable and sustainable development of the country’s mining industry.

The uncontrolled use of mercury in mining has led to serious consequences for both the environment and the population’s health in several Latin American countries. In Colombia, for instance, illegal mining in the departments of Chocó and Nariño has caused water contamination, deforestation, and security and violence problems in the region [78]. In Peru, illegal mining in the Madre de Dios region has resulted in contamination of the Amazon River and deforestation of large forest areas [79]. In addition, countries such as Indonesia, Tanzania, and Ghana, have reported the impact of mining activities on the environment and their negative effects on the surrounding populations. Table 4 summarizes some cases of contamination. Therefore, integrating research and policy to effectively address global and local mercury contamination and its health impacts is essential. Research findings must be promptly translated into appropriate preventive measures and human exposure monitoring programs through biomonitoring.

Given the above, it is crucial for the State of Ecuador to implement adequate sustainable mining practices and environmental regulations to minimize mercury releases and mitigate their impact on human health and the environment. Moreover, understanding the extent of contamination and developing effective remediation and risk management strategies require proper monitoring and assessment of the mercury levels in Ecuador’s coastal provinces. Responsible mining practices could include the use of clean and efficient technologies for mineral extraction, the implementation of restoration practices for degraded areas after mining operations closure, waste generation minimization practices such as reducing mercury use, and increased community participation, including consultation and prior informed consent of the population before initiating any mining activity. It should be noted that each situation is unique and requires specific solutions adapted to the circumstances of each case.

One of the limitations affecting many mercury abatement programs lies in the lack of consideration of the socio-technical complexity of the challenges associated with mercury in the mining industry. This is evidenced by focusing exclusively on technical aspects or educational strategies, without integrating the totality of the complex aspects inherent to mining [12,83]. In addition, findings related to non-cancer risk in the field of public health research have important implications for the development of policies and strategies aimed at reducing such risk and promoting the health and well-being of populations. When it comes to addressing noncancer risk, it is critical to consider a number of factors, ranging from exposure to environmental chemicals and pollutants to lifestyle behaviors and socioeconomic conditions. Public health policies and strategies must be based on sound and up-to-date evidence, including not only the identification of risk factors, but also an understanding of the underlying mechanisms and long-term effects on human health.

## 5. Conclusions

The findings presented in this paper provide relevant information about the presence of Hg in various rivers in five provinces of the northern Ecuadorian coastal area. In this sense, public policies must be established to limit the population’s exposure to Hg in the highest risk areas. Furthermore, it is essential to educate the population about the problems and risks of using Hg in mining, since the risk of developing systemic diseases is not limited to mining workers but to the population in general. In this context, decision makers must implement efficient solutions to the innumerable challenges posed by gold mining on the Ecuadorian coast.

Hg concentrations in surface water were evaluated and the potential health risk to the inhabitants of the area was determined. The results showed that at least 63% of the samples met the water quality standards for the protection of aquatic life according to current Ecuadorian regulations; the other 37% were below the LoD. On the other hand, the possibility of experiencing negative effects on human health due to Hg exposure was below the permissible limits for both adults and children in the residential setting according to the probabilistic risk analysis. But in the recreational setting, children present risk values above the safe exposure threshold. In contrast, according to the deterministic method, both receptors presented risk values higher than the recommended limit in the provinces of El Oro and Esmeraldas. Additionally, this study noted areas that exhibit potential systemic risk, especially for children, due to ingestion of water from surface water sources. These findings highlight the importance of incorporating a spatial distribution approach when assessing social and ecological effects in the area. This would enable more efficient management of natural resources and increased safeguarding and medical care for people exposed to Hg contamination.

Additional research is needed on contamination caused by human activities in the coastal region to analyze the amount and availability of mercury, methylmercury, and other potentially hazardous elements in various aspects, such as sediments, soils, food, and living organisms. This would allow for an expanded assessment of human health hazards and more effective management. It is also crucial to examine the quantity and availability of Hg, especially in areas where gold mining is taking place. Furthermore, it is important to evaluate the temporal and spatial evolution of pollutants in different periods, namely the rainy season and the dry season, since precipitation affects the possible dilution processes of pollutants. We believe that the findings of this study could be useful for the formulation of participatory policies and strategies related to water management and mitigation of pollution caused by human activities. Additionally, these results could serve as a starting point for addressing risks in the coastal region of Ecuador and in other areas of the country facing problems related to contamination by heavy metals and other pollutants. Finally, the participation and awareness of the population is essential for the success of any policy or strategy focused on controlling pollution and safeguarding human health and the environment.

Finally, it is important to address methodological limitations that may have influenced data collection and analysis. For example, the lack of access to certain coastal areas, the availability of specific sampling technologies, or the accuracy of analytical methods used to detect the presence of contaminants such as mercury and methylmercury. In addition, it would be beneficial to expand the scope of the study to include a more detailed analysis of the potential effects of pollution on marine biodiversity and coastal ecosystems. This could involve assessment of impacts on specific species, as well as analysis of trophic interactions and bioaccumulation of contaminants in the food chain. In terms of future lines of research, it would be valuable to further explore mitigation and remediation strategies that could be applied to reduce pollution in the coastal region. This could include the development of wastewater treatment technologies, the implementation of sustainable agricultural practices, and the promotion of safer alternatives for the extraction of minerals such as gold.

## Figures and Tables

**Table 1:** Parameters used for the risk assessment.

Parameter	Point value	Distribution
EF_residentil_ (day/year) ^a^	350	Triangular 345 (180–365)
EF_recreational_ (day/year) ^a^	120	Triangular 120 (26–260)
ET_residential_ (hour/event) ^b^	0.22	-
ET_recreational_ hour/event) ^a^	2.6	Triangular 2.6 (0.5–6)
IR_residential_ (L/day) ^a^	A = 2.04; C = 1.28	-
IR_recreational_ (L/event) ^c^	A = 0.053; C = 0.090	-
ED (year) ^b,d^	A = 30; C = 6	A = Lognormal 11.36 ± 13.72; C = Uniform 1–6
SA (cm^2^) ^b,e,f^	A = 23,000; C = 7280	A = Normal 18,400 ± 2300; C = Normal 6800 ± 600
Bw (kg) ^e,f^	A = 72; C = 15.6	A = Normal 72 ± 15.9; C = Normal 15.6 ± 3.7

A = adult; C= children; ^a^ [44] ^b^ [63]; ^c^ [64]; ^d^ [65]; ^e^ [66]; ^f^ [67].

**Table 2:** Mercury concentration in several provinces of the coast of Ecuador.

Province	Canton	Parish	River	SW (mg/L)	Latitude	Longitude
Esmeraldas	Mira	Jijon Caamaño	Santiago	*	0.852715	−78.778949
	Mira	Jijon Caamaño	Santiago	0.0042	0.877089	−78.797644
	Mira	Jijon Caamaño	Santiago	0.0032	0.925531	−78.863571
	Mira	Jijon Caamaño	Santiago	*	0.976836	−78.865442
	Mira	Jijon Caamaño	Cayapas	0.0017	0.807205	−78.929447
	Mira	Jijon Caamaño	Cayapas	0.0023	0.853356	−78.965355
	Mira	Jijon Caamaño	Cayapas	*	0.865573	−78.95687
	Mira	Jijon Caamaño	Cayapas	0.0007	0.947456	−78.995308
	Tulcan	El Chical	Esmeraldas	0.0013	0.456628	−79.395677
	Tulcan	El Chical	Esmeraldas	0.0045	0.539822	−79.447082
	Tulcan	El Chical	Esmeraldas	0.0015	0.626863	−79.53003
	Mira	Jijon Caamaño	Esmeraldas	0.0009	0.764589	−79.587814
Santo Domingo de los Tsáchilas	Santo Domingo	Alluriquin	Pilaton	*	−0.348695	−78.846268
	Santo Domingo	Alluriquin	Pilaton	*	−0.331799	−78.870037
	Santo Domingo	Alluriquin	Pilaton	*	−0.329653	−78.912094
	Santo Domingo	Alluriquin	Pilaton	*	−0.315835	−78.953979
Los Ríos	Valencia	Valencia	Mangulita	*	−0.874314	−79.242664
	Valencia	Valencia	Mangulita	*	−0.853437	−79.262112
	Valencia	Valencia	Mangulita	0.0007	−0.844426	−79.285115
	Valencia	Valencia	Mangulita Grande	*	−0.82739	−79.269834
	Valencia	Valencia	Mangulita Grande	*	−0.843417	−79.284462
	Valencia	Valencia	Mangulita	0.0023	−0.858074	−79.302015
	Ventanas	Ventanas	Oncebí	*	−1.355132	−79.302802
	Ventanas	Ventanas	Oncebí	0.0008	−1.357324	−79.361532
	Ventanas	Ventanas	Oncebí	*	−1.364625	−79.403852
Guayas	General Antonio Elizalde	General Antonio Elizalde	Chimbo	*	−2.203143	−79.126622
	General Antonio Elizalde	General Antonio Elizalde	Chimbo	*	−2.194694	−79.203102
	General Antonio Elizalde	General Antonio Elizalde	Chimbo	*	−2.185331	−79.318185
	General Antonio Elizalde	General Antonio Elizalde	Chimbo	*	−2.203569	−79.417346
El Oro	Pasaje	Pasaje	Jubones	0.0054	−3.314675	−79.699293
	Pasaje	Pasaje	Jubones	0.0015	−3.319784	−79.717638
	Pasaje	Pasaje	Jubones	0.0009	−3.323668	−79.760903
	Pasaje	Pasaje	Jubones	0.0023	−3.316602	−79.800791
	El Guabo	El Guabo	Jubones	0.0031	−3.262909	−79.870873
	El Guabo	El Guabo	Jubones	0.0065	−3.1754	−79.920519
	El Guabo	Río Boníto	Fermin	0.0042	−3.101681	−79.70762
	El Guabo	Río Boníto	Fermin	0.0009	−3.077169	−79.751994
	El Guabo	Río Boníto	Fermin	0.0007	−3.062427	−79.77122
	Portovelo	Portovelo	Amarillo	*	−3.679205	−79.579608
	Portovelo	Portovelo	Amarillo	0.0043	−3.698166	−79.594988
	Portovelo	Portovelo	Amarillo	0.0054	−3.711047	−79.606398
	Portovelo	Portovelo	Amarillo	0.0076	−3.727815	−79.633878
	Portovelo	Portovelo	Amarillo	0.0083	−3.745469	−79.642244
	Portovelo	Portovelo	Amarillo	0.0007	−3.764319	−79.646244
	Zaruma	Huertas	Calera	*	−3.661056	−79.642665
	Zaruma	Huertas	Calera	0.0098	−3.672964	−79.637725
	Zaruma	Malvas	Calera	0.0043	−3.684464	−79.646006
	Zaruma	Malvas	Calera	0.0008	−3.701343	−79.635028
	Portovelo	Portovelo	Calera	0.0009	−3.727677	−79.635287
	Chaguarpamba	Chaguarpamba	Pindo	*	−3.750791	−79.610629
	Chaguarpamba	Chaguarpamba	Pindo	0.0051	−3.768343	−79.645856
	Chaguarpamba	Chaguarpamba	Pindo	0.0897	−3.781962	−79.681651
	Chaguarpamba	Chaguarpamba	Pindo	0.0965	−3.830605	−79.719776
	Chaguarpamba	Chaguarpamba	Pindo	0.0952	−3.83492	−79.752066

* <LD.

**Table 3:** Deterministic HQ and HI (p95) Hg exposure for recipients by province.

Province	Risk	Residential Scenario		Recreative Scenario	
		Adults	Children	Adults	Children
El Oro	HQ_ingestion_	**8.52 × 10** **^0^**	**2.47 × 10^+1^**	6.90 × 10^−3^	5.40 × 10^−2^
	HQ_dermal_	3.02 × 10^−1^	4.41 × 10^−1^	**1.03 × 10^0^**	**1.50 × 10^0^**
	HI	**8.82** **× 10^0^**	**2.51 × 10^+1^**	**1.04 × 10^0^**	**1.55 × 10^0^**
Esmeraldas	HQ_ingestion_	3.94 × 10^−1^	**1.14 × 10^0^**	3.18 × 10^−4^	2.49 × 10^−3^
	HQ_dermal_	1.40 × 10^−2^	2.04 × 10^−2^	4.73 × 10^−2^	6.90 × 10^−2^
	HI	4.08 × 10^−1^	**1.16 × 10^0^**	4.76 × 10^−2^	7.15 × 10^−2^
Los Ríos	HQ_ingestion_	1.54 × 10^−1^	4.46 × 10^−1^	1.25 × 10^−4^	9.76 × 10^−4^
	HQ_dermal_	5.46 × 10^−3^	7.97 × 10^−3^	1.85 × 10^−2^	2.71 × 10^−2^
	HI	1.59 × 10^−1^	4.54 × 10^−1^	1.86 × 10^−2^	2.81 × 10^−2^
Santo Domingo de los Tsáchilas	HQ_ingestion_	2.26 × 10^−2^	6.56 × 10^−2^	1.83 × 10^−5^	1.44 × 10^−4^
	HQ_dermal_	8.02 × 10^−4^	1.17 × 10^−3^	2.73 × 10^−3^	3.98 × 10^−3^
	HI	2.34 × 10^−2^	6.68 × 10^−2^	2.75 × 10^−3^	4.12 × 10^−3^
Guayas	HQ_ingestion_	2.26 × 10^−2^	6.56 × 10^−2^	1.83 × 10^−5^	1.44 × 10^−4^
	HQ_dermal_	8.02 × 10^−4^	1.17 × 10^−3^	2.73 × 10^−3^	3.98 × 10^−3^
	HI	2.34 × 10^−2^	6.68 × 10^−2^	2.75 × 10^−3^	4.12 × 10^−3^

Values in bold exceed the safe exposure threshold.

**Table 4:** Summary of mercury concentrations in sites with mining influence.

Country	Media	Concentration Range (Min–Max)	Reference
Indonesia	Air	2096–3299 ng/m^3^	[80]
Tanzania	Surface water	0.01–0.07 µg/L	[81]
	Sediments	0.10–0.66 mg/kg	
Ghana	Waste soils	0.56–5.27 mg/kg	[82]
	Active mining	9.1–409.3 mg/kg	

## Data Availability

The data presented in this study are available on request from the corresponding author.
